# SARS-CoV-2 Vaccination in Children and Adolescents—A Joint Statement of the European Academy of Paediatrics and the European Confederation for Primary Care Paediatricians

**DOI:** 10.3389/fped.2021.721257

**Published:** 2021-08-23

**Authors:** Łukasz Dembiński, Miguel Vieira Martins, Gottfried Huss, Zachi Grossman, Shimon Barak, Christine Magendie, Stefano del Torso, Hans Jürgen Dornbusch, Artur Mazur, Katarzyna Albrecht, Adamos Hadjipanayis

**Affiliations:** ^1^The European Academy of Paediatrics, Brussels, Belgium; ^2^Department of Pediatric Gastroenterology and Nutrition, Medical University of Warsaw, Warsaw, Poland; ^3^Young European Academy of Paediatrics, Brussels, Belgium; ^4^Pediatrics Department, Cova da Beira University Hospital Centre, Covilha, Portugal; ^5^The European Confederation of Primary Care Paediatricians, Lyon, France; ^6^Kinder-Permanence Spital Zollikerberg, Zollikerberg, Switzerland; ^7^Adelson School of Medicine, Ariel University, Ariel, Israel; ^8^Maccabi Health Services, Tel Aviv, Israel; ^9^Tel Aviv Sourasky Medical Center, Dana-Dwek Children's Hospital, Tel Aviv, Israel; ^10^Association Française de Pédiatrie Ambulatoire, Talence, France; ^11^Childcare Worldwide, Padova, Italy; ^12^Department of Pediatrics and Adolescent Medicine, Medical University of Graz, Graz, Austria; ^13^Medical Faculty, University of Rzeszow, Rzeszów, Poland; ^14^Department of Pediatric, Hematology and Oncology, Medical University of Warsaw, Warsaw, Poland; ^15^School of Medicine, European University Cyprus, Nicosia, Cyprus; ^16^Paediatric Department, Larnaca General Hospital, Larnaca, Cyprus

**Keywords:** adolescents, children, COVID-19, infectious diseases prevention, pandemic, vaccines

## Abstract

Stopping the COVID-19 pandemic and its socio-economic consequences is only possible with a multifaceted strategy, including mass vaccination. Studies have been conducted mainly in adults, and data on the pediatric population is relatively limited. However, it appears that vaccination in children and adolescents is highly effective and safe. Despite the apparent benefits of vaccinating this age group, there are some medical and ethical concerns. Based on the above considerations, the European Academy of Paediatrics (EAP) and the European Confederation of Primary Care Pediatricians (ECPCP) assessed the current situation and presented recommendations for international and national authorities, pediatricians, and pediatric societies regarding vaccination against SARS-CoV-2 in children and adolescents.

## Introduction

Since its initial outbreak in Wuhan, China, in December 2019, the novel coronavirus disease (COVID-19) has affected over 180 million persons and caused more than 3.9 million deaths worldwide (1). Despite efficacious attempts at containing the disease, such as home confinement, mandatory use of facial masks, and social distancing, COVID-19 remains a significant cause for morbidity and mortality globally. The reported incidence of this disease in children is lower than in adults, accounting for ~5% of total cases, according to the Centers for Disease Control and Prevention (2). Although less prevalent in children, the socio-economic, educational, and public health impact of COVID-19 on our society has severely affected children and adolescents' physical, intellectual, and emotional development (3). It is estimated that ~1.5 billion young people worldwide were in mandatory confinement at home, and this situation has negatively influenced their health and social functioning (4).

## Covid-19

Children and adolescents appear to have a milder form of the disease than adults but remain susceptible to infection and severe manifestations across all ages (5). Considered primarily as a respiratory disease, COVID-19 manifests in children most commonly as a flu-like illness with fever and cough (6). Of those requiring hospital admission, about one-third will be admitted to intensive care (7). Children with previous respiratory or cardiovascular conditions are thought to be at higher risk of the severe acute respiratory syndrome coronavirus-2 (SARS-CoV-2) infection. A recent systematic review corroborated this notion and highlighted a higher infection susceptibility in patients with asthma or renal malformation, although more substantial evidence is warranted (8).

## MIS-C

Moreover, a newly described syndrome referred to as multisystem inflammatory syndrome in children (MIS-C), or pediatric inflammatory multisystem syndrome (PIMS-TS), has been observed shortly after COVID-19 infection in a subset of pediatric patients. Studies characterizing MIS-C/PIMS-TS are fast emerging. Co-existing illnesses have been described in a few multicentric studies ranging from 3% to as much as 25%, with great variability (9, 10). From these respiratory diseases, obesity and cardiovascular conditions were the most frequently noted (10). Other co-existing neuromuscular, oncologic, immunosuppressive, autoimmune, and genetic conditions have also been noted, though much less frequently (10–12). On the other hand, complications following MIS-C/PIMS-TS may include myocardial dysfunction, shock, and respiratory failure requiring intensive care (13).

## Long Covid-19

Although SARS-CoV-2 infection in children is rarely severe, the long-term consequences of COVID-19 are not yet fully understood. Preliminary evidence shows that long-standing effects of COVID-19 may be seen up to 6 months after infection, namely fatigue, muscle and joint pain, insomnia, respiratory problems, and palpitations, difficult to distinguish from “pandemic blues” or other conditions frequently seen in teenagers (14). Altogether, this evidence highlights the need for active public health and policy strategies to mitigate the pandemic's impact on children's health. In addition to the direct health benefits of immunization against SARS-CoV-2, an effective vaccination could reduce the marked social impact of COVID-19 in children (15).

## Clinical Trials

Based on the results of clinical trials and the effects of mass vaccination against COVID-19, it appears that the immunogenicity of available vaccines is as high as 90–95% (16–19). However, this experience applies mainly to the adult population because the results published so far refer only to studies in which participants were ≥16 years of age (15, 17, 18).

Typically, enrolling children and adolescents in clinical trials follow adult trials for safety, ethical, and organizational reasons (20). However, in aiming at an effective control of the COVID-19 pandemic, the lack of reliable data on such a large population as children and adolescents may endanger not only themselves but also entire families and compromise the timely achievement of population immunity (21). Clinical trials in children and adolescents are still ongoing (22). However, the first results for the Pfizer-BioNTech vaccine in children ≥ 12 years of age are more than promising—vaccine efficacy reached 97–100%—which has already encouraged several authorities to approve the vaccine in this population (23–25). Studies in younger children have also just begun, but the results will only be available in several months (26, 27). Because there is still a need for reliable data, it is essential to accelerate trials on the immunogenicity and safety of SARS-CoV-2 vaccines in children.

## Apparent Vaccination Benefits

The apparent advantage of vaccinating children and adolescents is to protect them from COVID-19. Although the course of the disease in children is often mild or even symptomatic, severe cases have been reported (28). Moreover, as in adults, there is some evidence that a considerable number of children who contract the virus have at least one symptom lasting more than 4 months. This long COVID-19 or post-COVID-19 syndrome, although, due to the quality of the data, poorly proven, may impair children's daily activities, development, and have a lifelong health impact (29, 30).

Hypothetically, vaccines may also prove effective in preventing MIS-C/PIMS-TS in younger children, although this has yet to be established because a potential reverse correlation cannot be ruled out at this point.

Existing data on the role of children in the transmission of SARS-CoV-2 and mucosal carriers are not entirely conclusive (31, 32). They do not appear to play a major role in the transmission of the virus. However, due to the intensity of their contacts and their significant number, their role should not be underestimated. Vaccination optimization models also do not provide a definite answer. Vaccination of older adults with comorbidities should be an undeniable priority. However, assuming that not all adults will be vaccinated (also as a result of their choice), vaccinating children and adolescents may increase the chance of achieving herd immunity (33–35).

Vaccinating children and adolescents should also reduce doubts about their return to school. For more than a year, the forced lockdown of children at home has caused the accumulation of educational, developmental, and psychological problems (4). A faster return to regular school learning and peer interaction is vital for reversing these trends.

Providing immunity to children will also encourage the return of regular pediatric care, including vaccination against other infectious diseases, which were severely disrupted during the pandemic and lockdown (36).

Moreover, home care for a sick child or for those forced to be at home during the closure of kindergartens and schools has socio-economic consequences related to the absence of caregivers from work. Vaccinating children and adolescents could potentially prevent these consequences (37).

## Immunogenicity and Effectiveness

One of the main concerns regarding vaccinating children and adolescents against SARS-CoV-2 are the lack of sufficient data on vaccine-induced long-term immunogenicity in children and, even more critical—safety data (38). However, the first results on the short-term effects of vaccines are promising, and there will likely be more in the coming months (23, 24, 39, 40). Characteristics and age of approval of select COVID-19 vaccines are presented in Table 1 (41, 42);^1^.

**Table 1:** Characteristics and age of approval of select COVID-19 vaccines (*approved in the USA).

**Name**	**Company/developer**	**Platform**	**Doses and intended interval**	**Efficacy against symptomatic COVID-19**	**Age of approval**	**Clinical Trials*ClinicalTrials.gov (ages)***	**Clinical trial phase and current status**
BNT162b2	Pfizer/BioNTech	mRNA	2 doses 3 weeks apart	95%	>12 years*	NCT04816643 (6 months−11 years)	Phase 1/2 - recruiting
mRNA-1273	Moderna	mRNA	2 doses 4 weeks apart	94%	>12 years*	NCT04796896 (6 months−11 years)	Phase 2/3 - recruiting
Ad26.COV2.S	Janssen/Johnson & Johnson	Replication-incompetent adenovirus 26 vector	1 dose	66%	>18 years	NCT04535453 (12–17 years)	Phase 2 - active, not recruiting
ChAdOx1 nCov-19/AZD1222	AstraZeneca/University of Oxford/ Serum Institute of India	Replication-incompetent chimpanzee adenovirus vector	2 doses, 8-12 weeks apart (WHO recommendation)	70%	>18 years	–	–
NVX-CoV2373	Novavax	Recombinant protein	2 doses 3 weeks apart	89%	>18 years	NCT04611802 (12–17 years)	Phase 3 - recruiting
Gam-COVID-Vac (Sputnik V)	Gamaleya Institute	Replication-incompetent adenovirus 26 and adenovirus 5 vectors	2 doses 3 weeks apart	92%	>18 years	NCT04954092 (12–17 years)	Phase 2/3 - recruiting

## Safety

Regardless of the undeniable potential benefits of vaccinations, the question remains whether they are safe in children and adolescents. As with immunogenicity, the short-term safety assessment of the vaccine has been positive (40). Adverse events were reported only in 5 out of 1,131 vaccinated children, and none of these were related to the study intervention (23, 24). However, no data on long-term effects are available yet, and rare age-specific undesirable effects may only emerge with increasing vaccination coverage.

There are also reports of a possible coincidence of SARS-CoV-2 vaccination and myocarditis (43–45). The scale of this potential adverse event does not appear to be significant, as the Centers for Disease Control and Prevention (CDC) has recorded more than 1,000 reported cases, with nearly 180 million US citizens vaccinated (46). Myocarditis seems to affect primarily male adolescents aged 16 years or older after the second dose of the vaccine, and recovery occurs relatively quickly. However, in the case of such a sensitive issue as the vaccination of children, the safety aspect is crucial, and requires thorough investigation.

Furthermore, most of the available data are manufacturers' announcements and preliminary results that have not yet passed the critical review preceding publications. The results should not be generalized to all SARS-CoV-2 vaccines either, because for now, we only have data mainly on one of them. However, as stated by the CDC: currently, the benefits of vaccination outweigh the risks for COVID-19 vaccination in adolescents and young adults (47).

## Allocation

The second major dilemma in vaccinating youth is the ethical concern related to vaccine allocation. Vaccinating children and adolescents who are less exposed to the severe course of COVID-19 consumes vaccine resources that, under optimal conditions, could be used to immunize high-risk groups. This aspect is particularly important for developing countries where access to healthcare is unequal and vaccine supply is limited (48–50). Equitable vaccine distribution between high- and low-income countries will be crucial for suppressing virus circulation and the emergence of new variants of concern and eventually halt the asynchronous waves of the pandemic. However, it should be noted that the potential use of vaccines for vaccination of children and adolescents in Europe will be incomparably lower than the needs of developing countries and the effect limited by difficult distribution logistics.

## Caregivers' Willingness

According to surveys, the willingness to vaccinate children is expressed by ~60–70% of caregivers, which agrees with earlier research showing that nearly 30% of respondents doubt whether to vaccinate themselves (51–54). In adolescents over 14 years of age, 76% of respondents declared their willingness to be vaccinated (55). However, there is a risk that as fear of the pandemic decreases, the percentage of those willing to be vaccinated will also decline; as was the case, e.g., with the measles vaccine (56). Therefore, it is crucial to encourage caregivers to vaccinate their children.

## Mandatory Vaccination

Although there is no doubt that SARS-CoV-2 vaccines should be as widely available as possible and financed from public funds, it is ethically controversial that vaccination should be mandatory for both adults and children (57, 58). In many European countries, vaccination against other infectious diseases has been compulsory for many years, which appears to impact vaccination coverage significantly (59). Such restrictions are often questioned, and there are some indications that the counseling encouraging voluntary vaccination and information campaigns are more effective in the longer term (60, 61). Nevertheless, the global threat of the COVID-19 pandemic may require more restrictive management, and this decision should remain with the national authorities.

## Current Practice

Despite the lack of extensive data on the immunogenicity of vaccination against SARS-CoV-2 in children and adolescents, these vaccinations appear to be safe (23, 24). Therefore, a growing number of countries is beginning to vaccinate children and adolescents. The United States of America, Canada, and the European Union have recently registered vaccines for children ≥ 12 years of age (62–64). In addition, the American Academy of Pediatrics, the Advisory Committee on Immunization Practices, and the Canadian Pediatric Society advocate for the vaccination of all children and adolescents older than 12 years (65–67). However, the United Kingdom is holding back this decision due to the lack of clear evidence about the safety and need of vaccination against SARS-CoV-2 in children (68). On the other hand, the European Center for Disease Prevention and Control stated that due to the limited benefits in children and adolescents, careful consideration of the epidemiological situation and vaccine uptake in older age groups should be given before targeting children and adolescents (69).

## Recommendations

The European Academy of Paediatrics (EAP) and the European Confederation for Primary Care Paediatricians (ECPCP) strongly believe that action is necessary to clarify the future of SARS-CoV-2 vaccination in children and adolescents at different levels.

### For International and National State, Health, and Education Authorities

- It is essential to clarify the ethical issues of allocating SARS-CoV-2 vaccination in relation to age groups, risk groups, and each country's level of development.
- Clinical trials on SARS-COV-2 vaccination in children and adolescents of all ages should be supported and accelerated to provide reliable data on immunogenicity, safety, and epidemiological effectiveness to control the pandemic.
- If these latter factors are demonstrated, children and adolescents' vaccination should be promoted and encouraged worldwide.
- It is necessary to establish a legal framework for the SARS-Cov-2 vaccination in children and adolescents.
- Once the above conditions are met, pediatricians and other child healthcare providers should be supported and encouraged to perform SARS-COV-2 vaccination in children and adolescents to ensure high coverage.
- Children suffering from underlying disease and their families should be granted priority access to vaccination promptly.

### For European and National Pediatric Societies

- Members should be regularly updated on all aspects of SARS-COV-2 vaccination in children and adolescents.
- There should be a continuous scientific discussion weighing the advantages and disadvantages of vaccinating children and adolescents against COVID-19.
- It is advisable to coordinate national and international pediatric societies to promote the vaccination of children and adolescents against SARS-COV-2.
- Tools should be developed to motivate healthcare workers, caregivers, and children during the vaccination campaign.
- National pediatric associations should be encouraged to develop national strategies of SARS-COV-2 vaccination integration into existing vaccination schedules.
- Primary healthcare should be supported in implementing vaccination against SARS-COV-2 in children and adolescents.
- National pediatric societies should support the EAP campaign “Vaccinate your child” to restore missed or delayed routine immunization of children.

### For Providers in the Primary Pediatric Care

- Efforts should be made to provide reliable information on SARS-CoV-2 vaccination to other healthcare professionals, caregivers, children, and adolescents to encourage them to be vaccinated. In addition, evidence-based materials for parents, children, and adolescents should be prepared.
- SARS-CoV-2 vaccination should be integrated into current child healthcare practices and vaccination schedules.
- Appropriate organizational support in vaccinating children and adolescents should be demanded from the community, local health, and educational authorities.

## Data Availability Statement

The original contributions presented in the study are included in the article/supplementary material, further inquiries can be directed to the corresponding author/s.

## Author Contributions

ŁD, MV, GH, ZG, SB, ST, AM, and AH: study design. ŁD, MV, GH, ZG, SB, CM, ST, HD, and KA: data collection. ŁD, MV, GH, ZG, SB, CM, ST, HD, AM, KA, and AH: data analysis and interpretation, manuscript preparation, and critical revision. All authors read and approved the final manuscript.

## Conflict of Interest

The authors declare that the research was conducted in the absence of any commercial or financial relationships that could be construed as a potential conflict of interest.

## Publisher's Note

All claims expressed in this article are solely those of the authors and do not necessarily represent those of their affiliated organizations, or those of the publisher, the editors and the reviewers. Any product that may be evaluated in this article, or claim that may be made by its manufacturer, is not guaranteed or endorsed by the publisher.

## Footnotes

^1^Available online at: https://clinicaltrials.gov (accessed July 19, 2021).
